# Magnetic Field Sensing Using Tapered Small-Core Optical Fibre Surrounded by Different Concentrations of Magnetic Fluid

**DOI:** 10.3390/s22218536

**Published:** 2022-11-05

**Authors:** Rahul Kumar

**Affiliations:** 1 Perth College, University of Highlands and Islands, Perth PH1 2NX, UK; rahul.kumar.perth@uhi.ac.uk; 2 Faculty of Engineering and Environment, Northumbria University, Newcastle upon Tyne NE1 8ST, UK

**Keywords:** optical fibre sensor, magnetic field sensing, tapered small-core, magnetic fluid

## Abstract

In this paper, a high-sensitivity magnetic field sensor based on a single-mode–tapered small-core–single-mode (STSCS) optical fibre structure is investigated. The tapered small-core section of STSCS is surrounded by magnetic fluid (MF) containing ferromagnetic particles (FMPs) of different concentrations. The FMPs align themselves along the magnetic field, depending on the strength of the magnetic field. This alignment of FMPs changes the refractive index around the tapered small-core section, which in turn changes the output spectral response of the STSCS optical fibre structure. The change in spectral response is then calibrated for sensing the magnetic field strength. This paper also investigates the effect of both the taper waist diameter of the STSCS optical fibre structure and the concentration of MF surrounding it on the magnetic field sensitivity. The maximum sensitivity demonstrated in this paper is 0.46 nm/mT for a taper waist diameter of 10 μm surrounded by 1.22% FMPs in the MF. The magnetic sensor demonstrates reversible results, and its effects on the orientation of the magnetic field along the X–Y, X–Z and Y–Z axes are also investigated, which suggest that the sensor is capable of vector magnetic field measurement.

## 1. Introduction

Optical fibre magnetic sensors based on magnetic fluids (MFs) have recently gained significant interest due to their magneto-optic properties, such as their field-dependent refractive index (RI), optical birefringence, and transmission [1,2]. The most widely used MFs are water- and oil-based, in which the ferromagnetic nanoparticles are distributed evenly throughout the volume in either water or oil. In the past, many magnetic field sensors have been modified by combining different structures of optical fibre with magnetically susceptible fluids, which were either in an aqueous solution or a polymer matrix around the optical fibre. For example, a magnetic sensor based on cladding etched fibre Bragg gratings (FBGs) [3] and tilted FBGs [4] interacting with MFs was proposed by Dai et al. and Zheng et al., respectively. However, the sensors were reported to have a sensitivity as low as 10 pm/mT due to the low interaction between the fundamental core mode and higher-order cladding modes. Another magnetic field sensor based on a Sagnac interferometer, with a higher sensitivity of ~0.167 nm/mT, was reported by Zu et al. The sensor used a free-space MF film with two collimators, but it has a more complex structure [5].

Microfibres are sensitive to changes in external RI because of evanescent field interaction with the surrounding medium. Several microfibre-based magnetic field sensors have been reported with higher magnetic field sensitivities. For example, an optical microfibre mode interferometer coated with MF was demonstrated to have a sensitivity of −0.293 nm/mT [6]. Another magnetic field sensor, with an asymmetric taper with a waist diameter of 45 μm, immersed in MF was demonstrated to have a sensitivity of 0.162 nm/mT [7]. Luo et al. [8] demonstrated a magnetic field sensitivity of 1.918 nm/mT within a narrow range of a weak magnetic field up to 300 mT using an optical microfibre coupler (OMFC) surrounded by MF. A highly sensitive magnetic field sensor using OMFC combined with a MF in a Sagnac loop, formed using polarization-maintaining fibre, was demonstrated by Wei et al. [9]. The sensor had a sensitivity of −0.488 nm/mT within the range of 0–200 mT, but due to small taper waist diameter, the sensor, based on an OMFC structure suffered from the disadvantage of low stability. Recently, a bidirectional magnetic field sensor using microfibre wrapped around a micro-bottle filled with MF to form a resonator was demonstrated for magnetic field sensing by Zhao, et al. [10]. The proposed sensor had sensitivities of 98.23 pm/mT and −304.8 pm/mT, for perpendicular and parallel magnetic fields, respectively. Another microfibre suspended in a magnetorheological fluid film was proposed by Kumar et al. [11], with a maximum sensitivity of ~16.4 pm/mT over a large magnetic field range of 0–567 mT. Zhang et al., proposed a magnetic field sensor based on nonadiabatic tapered microfibre cascaded with FBG for the simultaneous measurement of magnetic field and temperature. For a magnetic field range of 0–18 mT, the maximum sensitivity demonstrated was 1.159 nm/mT [12].

Optical fibre heterostructures and special fibre structures have also been demonstrated for magnetic field sensing using MFs. For example, single-mode–hollow core–single-mode (SHS) optical fibre immersed in MF demonstrated a sensitivity of −1.7 nm/mT [13]. However, due to an irregular spectral response vs. magnetic field variation, it only had a narrow measurement range. A spherical bulb structure on a single-mode fibre coated with MF [14], a D-shaped fibre structure immersed in MF [15], and a peanut-shaped structure cascaded with long-period grating [16] have also been demonstrated for magnetic field sensing. However, these techniques have relatively low sensitivity.

This paper proposes and experimentally demonstrates a high-sensitivity single-mode–tapered small-core–single-mode (STSCS) [17] optical fibre structure encapsulated in MF for magnetic field detection. The paper also analyses the effect of the taper waist diameter and the concentration of FMPs in MF on the sensitivity. The influence of magnetic field orientation on the proposed sensor is also investigated in this paper. Such optical fibre sensors have the added advantage of good stability, compact size, immunity to electromagnetic interference, and remote-sensing capabilities [18] in comparison to their electronic counterpart. Therefore, they can be used in hazardous locations, including nuclear power plants, where electronic sensors would be unsuitable.

The remainder of the paper is organised as follows: Section 2 explains the theory and operating principle of magnetic fluid and STSCS sensors. Section 3 describes the experimental setup, fabrication technique, and working of the proposed STSCS optical fibre structure encapsulated in MF. Section 4 describes the results based on the taper waist diameter of the small-core and the MF concentration that were optimised for improving the magnetic field sensitivity. The effect of magnetic field orientation on the proposed sensor is also demonstrated in this section. Finally, Section 5 presents our conclusion.

## 2. Theory

### 2.1. Operating Principle of Magnetic Fluid

MFs are stable colloidal solutions consisting of ferromagnetic particles (FMPs) dispersed in a liquid carrier medium [19]. In the absence of a magnetic field, the FMPs are distributed randomly within the carrier medium. However, when a magnetic field is applied, the FMPs undergo Brown and Neel relaxation [20], thus forming a chain along the direction of applied magnetic field. Yang et al., in 2002 [21], measured the RI of a MF ($n_{\mathrm{MF}}$) under different magnetic field intensities (H) using the total reflection technique. The change in $n_{\mathrm{MF}}\;$at different Hs and temperatures (Ts), because of chain or column formation can be calculated by the following Langevin function [22]:

(1) $$n_{\mathrm{MF}}=\left[n_{s}-n_{0}\right]\left[coth\left(\alpha \frac{H-H_{c,n}}{T}\right)-\frac{T}{\alpha \left(H-H_{c,n}\right)}\right]+n_{0}\;for\;H>H_{c,n}$$

where

$H_{c,n}$: critical magnetic field intensity, which starts to change the RI of the MF,
$n_{0}$: RI of the MF under fields lower than $H_{c,n}$,
$n_{s}$: saturated value of the RI of the MF, and
α: fitting parameter.

### 2.2. Operating Principle of STSCS Sensor

**Figure 1:**
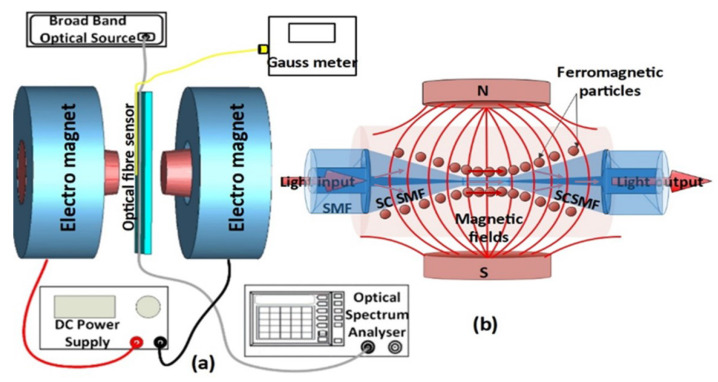
Schematic diagram of (**a**) experimental setup and (**b**) tapered small-core single-mode optical fibre sensor surrounded by magnetic fluid, for magnetic field sensing.

The core diameter of the small-core single-mode (SCSMF) optical fibre (SM-450, Thorlabs) is smaller than that of traditional single-mode fibre (SMF28). As a result of the core diameter mismatch, the SMF28–SCSMF–SMF28 sensor structure that operates at longer wavelengths supports higher-order cladding modes. Tapering the SCSMF section into a biconical structure, as shown in Figure 1b, supports further higher-order modes in the tapered waist region, which enhances the RI sensitivity of the sensor.

The sensing principle of the STSCS structure is based on the optical path difference between the core and cladding modes. The light entering the tapered SCSMF region from the SMF28 excites higher-order cladding modes in the tapered region. The fundamental core modes and the higher-order cladding modes couple together at the output SMF28, to form an interference pattern due to the difference in their propagation constants. The intensity from the output SMF28 can be expressed as [23]

(2) $$I=I_{core}+I_{clad}+2\sqrt{I_{core}I_{clad}}\cos\left(\Delta \varphi \right)$$

where $I_{core}$ and $I_{clad}$ are the intensities of the fundamental core mode and higher-order cladding mode, respectively, and Δφ represents the phase difference between the core and the cladding modes, which can be expressed as

(3) $$\Delta \varphi =\frac{2\pi }{\lambda }\Delta n_{eff}L$$

where λ represents the wavelength of the incident light, L is the uniform waist length, and $\Delta n_{eff}$ is the effective refractive index difference between the core and cladding mode, which is given by

(4) $$\Delta n_{eff}=n_{eff}^{core}-n_{eff}^{clad}\;(\Delta n_{eff}>0)$$

where $n_{eff}^{core}$ and $n_{eff}^{clad}$ are the effective refractive indices of the core and cladding mode, respectively.

According to (2), when Δφ is an odd multiple of π, the output light intensity is minimal. In Equation (2), the wavelength of the *m*-th order interference dip $\lambda_{m}$ can be expressed as

(5) $$\lambda_{m}=\frac{2\Delta n_{eff}L}{2m+1}$$

When the STSCS is surrounded by MF, the RI of the MF changes when an external magnetic field is applied, as explained above. An increase in the RI of the MF increases the $n_{eff}^{core}$ and $n_{eff}^{clad}$. However, the $n_{eff}^{core}$ increases by a much larger value than does the $n_{eff}^{clad}$ [24]. Therefore, a shift towards a longer wavelength is observed in the interference spectrum [12].

## 3. Experimental Setup

In this experiment, the STSCS optical fibre sensor structure was fabricated by tapering the SCSMF section of an SMF28–SCSMF–SMF28 using the traditional heat-pull technique [25]. To look at how the taper waist diameter affects the sensor’s sensitivity, three STSCSs with tapered waist diameters of 10, 15, and 20 μm were fabricated. For packaging the STSCS inside the glass capillary (inner diameter 1.15 ± 0.05 mm, outer diameter 1.5 ± 0.05 mm, and length ~5 mm), a sliding mechanism and additional set of fibre holders were used to ensure that the fibre remained straight inside the capillary. Once the tapered SC section was in the middle of the capillary tube, both the fibre and the capillary tube were fixed on a glass slide. The capillary tube was then filled with the desired concentration of MF using a flexible, fine-tip pipette, ensuring that no bubble or airgap was formed inside the capillary tube. The ends of the capillary tube were then sealed with sealing wax. Figure 1b shows the schematic diagram of the fabricated sensor. For this experiment, an oil-based MF with FMPs that averaged 10 nm in size was used. The original concentration of the MF contained, by volume, 5% FMPs, 10% surfactant/dispersant, and 85% carrier [26]. The MF was diluted to three different concentrations of 1.2%, 0.8%, and 0.4% of FMPs in order to study the effect of MF concentration on the magnetic field sensitivity of the fibre sensor.

Figure 1a shows a schematic diagram of the experimental setup used to test the magnetic sensors. The setup consists of an electromagnet, and the strength of its magnetic field could be controlled by varying the current flowing through its coil via a DC power supply. The actual value of the magnetic field strength was measured for reference using a Gauss metre, the probe of which was positioned halfway between two electromagnets close to the optical fibre sensor (about 1 mm apart). The STSCS’s taper waist region was placed near the middle of the magnetic poles, where the magnetic field density was maximal. To launch light into the optical fibre sensor, a broadband optical source (SLD1005S) was used, with the other end connected to an optical spectrum analyser (OSA) to analyse the sensor’s spectral response.

The FMPs are distributed randomly around the STSCS sensor in the absence of a magnetic field. When an external magnetic field is applied to the sensor, the FMPs orient themselves along the magnetic field lines, as shown in Figure 1b. The RI around the fibre sensor is altered by the alignment of the FMPs around the STSCS. As the magnetic field strength increases, more FMPs from the MF align along the magnetic field, further changing the RI around the fibre sensor. When the external magnetic field is removed, the FMPs revert to their initial random alignment.

## 4. Results and Discussion

### 4.1. Effect of MF Concentration and Reversibility

**Figure 2:**
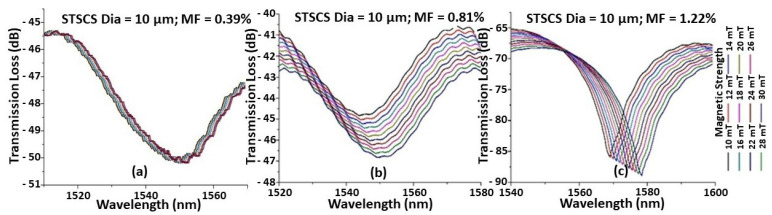
Example of spectral response observed while increasing the magnetic field around a 10 μm waist diameter STSCS optical fibre magnetic field sensor, surrounded by MF with (**a**) 0.39%, (**b**) 0.81%, and (**c**) 1.22% FMPs.

Figure 2 shows the spectral response observed for the STSCS with a tapered waist diameter of 10 μm encapsulated in MF containing (a) 0.39%, (b) 0.81%, and (c) 1.22% FMPs. It can be seen that, as the magnetic field strength increases, the spectral dip shifts monotonically towards longer wavelengths. This is due to the increased RI of the MF around the fibre sensor caused by the increase in the number of FMP alignments around the fibre sensor, as previously explained. For the same magnetic field strength, the STSCS encapsulated in a higher concentration of MF exhibits a larger spectral shift. This is due to the fact that higher concentrations of MF contain more FMPs, so for the same value of magnetic field strength, a larger number of FMPs will align near the STSCS optical fibre sensor, resulting in larger RI variations surrounding the sensor. Since the spectral responses were noisy, a 3 dB dip wavelength analysis was used to calculate the average position of the spectral dip.

**Figure 3:**
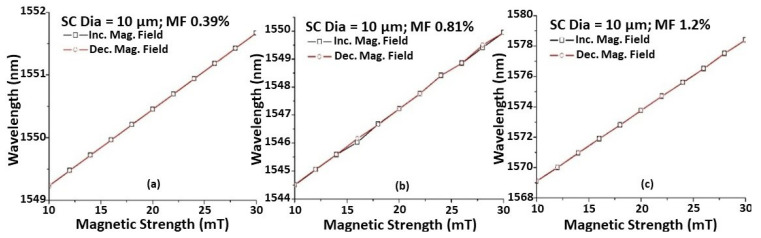
Wavelength shift caused by the change in magnetic field for STSCS of 10 μm tapered waist diameter encapsulated in (**a**) 0.39%, (**b**) 0.81%, and (**c**) 1.2% FMPs in MF.

Figure 3 shows the wavelength shift versus magnetic field strength. As explained previously, the sensitivity to the magnetic field increases with increasing MF concentrations for the same taper waist diameter of the STSCS. Each reading was taken 2 min after the magnetic field was applied to the sensor to ensure that the spectral response had stabilised, which could reduce the hysteresis effect and allow the FMPs to release residual magnetisation. It can also be seen that the wavelength shift for a change in magnetic field strength remains nearly constant while increasing and decreasing the magnetic field strength, demonstrating that the proposed sensor has good reversibility.

### 4.2. Effect of the Taper Waist Diameter of STSCS

**Figure 4:**
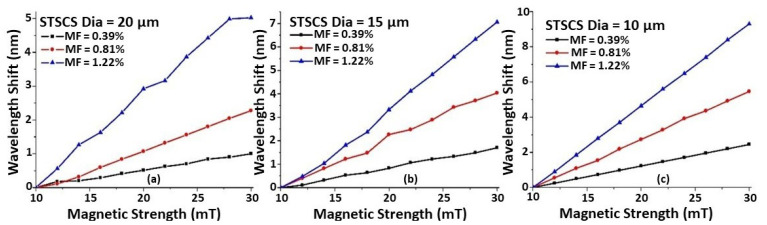
Wavelength shift versus magnetic field strength of STSCS with waist diameters of (**a**) 20 μm, (**b**) 15 μm, and (**c**) 10 μm, surrounded by different MF concentrations (0.39, 0.81, and 1.22% FMPs).

To study the effect of the taper waist diameter on magnetic field sensitivity, nine STSCS fibre sensors were fabricated with waist diameters of (a) 20 μm, (b) 15 μm, and (c) 10 μm, and they were surrounded by MFs of three different concentrations (0.39, 0.81, and 1.2% FMPs). Figure 4 shows the wavelength shift as a function of magnetic field strength for each of the nine sensors. It can be seen that, for all of the fabricated sensors, the wavelength shift shows good linearity with increasing magnetic field strength. As the taper waist diameter of the STSCS decreases from 20 μm to 10 μm, the rate of wavelength shift, which is the sensitivity of the fabricated STSCS optical fibre sensor, increases. This occurs because a smaller tapering diameter allows more of the evanescent field to interact with the surrounding medium, which increases the RI sensitivity of the STSCS fibre sensor. Figure 4 also shows that, for all three taper waist diameters, the sensitivity to magnetic field strength increases as the concentration of FMPs increases.

### 4.3. Sensitivity Comparison

**Figure 5:**
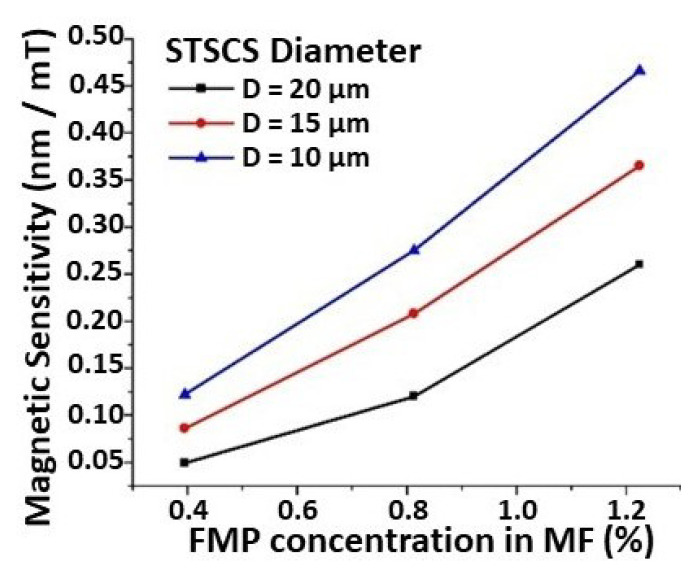
Magnetic sensitivity comparison for STSCS optical fibre sensors with 10, 15, and 20 μm diameters surrounded by 0.39, 0.81, and 1.22% FMPs in the MF.

Figure 5 shows the relationship between sensor sensitivity, taper waist diameter, and FMP concentration. The maximum observed sensitivity was 0.46 nm/mT for the STSCS with a taper waist diameter of 10 μm, surrounded by the MF with an FMP concentration of 1.22%. The lowest sensitivity observed was 0.049 nm/mT for the STSCS with a taper waist diameter of 20 μm surrounded by 0.39% FMPs in MF. The STSCS with a 10 μm taper waist diameter attained the highest set of magnetic field sensitivities using the three FMP concentrations (0.39, 0.81, and 1.22% FMPs), proving that smaller taper waist sensors have higher sensitivities. The numerical values of the sensitivities observed for all nine fabricated sensors with different STSCS waist diameters enclosed in different MF concentrations are listed in Table 1.

### 4.4. Effect of Magnetic Field Orientation

**Figure 6:**
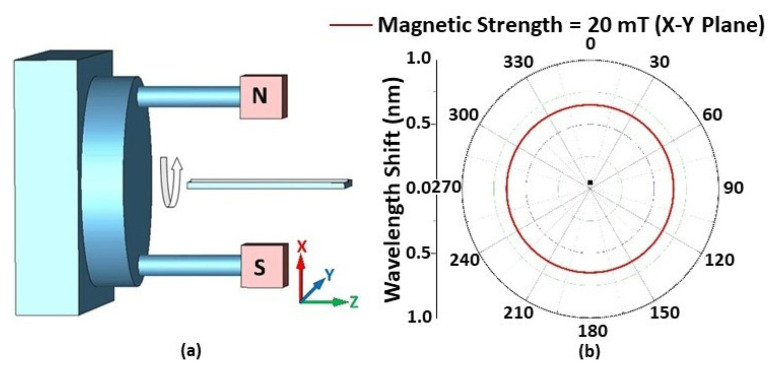
(**a**) The experimental setup used for measuring the angular magnetic field dependence in the X–Y plane, and (**b**) wavelength shift form λ_0_ when the rotation angle changes from 0° to 360° in the X–Y plane.

**Figure 7:**
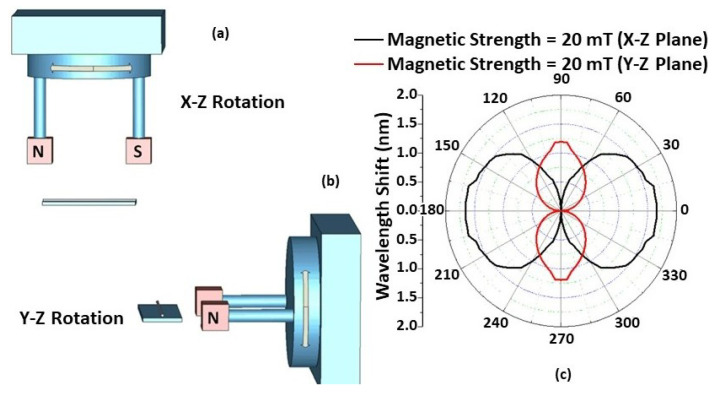
Schematic of the experimental setup used for measurements of the angular field dependence in (**a**) the X–Z and (**b**) the Y–Z plane; (**c**) Experimentally measured wavelength shift from λ_0_ versus from 0° to 360° in the X–Z and Y–Z planes for different magnetic field strength values.

The effect of magnetic field orientation on the performance of the STSCS-based magnetic sensor was also investigated using a fibre sensor with a 10 μm taper waist diameter and a 1.22% FMP concentration in the MF. A separate experimental setup was used for this purpose, consisting of two 20 mT permanent magnets that created a parallel and homogeneous magnetic field between them. The setup included a 360° rotating platform over which the two magnets were fixed, and the fibre sensor was held at an equal distance from the two magnets with the help of another holder. The orientation of the magnetic field applied to the sensor was changed by rotating the platform in 5° steps along the X–Y, X–Z, and Y–Z planes, as shown in Figure 6 and Figure 7. The rotation angle α is the clockwise angle between the direction of the STSCS axis and the normal line of the magnetic field. When the magnetic field axis is normal to the fibre axis, then α is considered to be 0°, while α is considered to be 90° when the magnetic field axis is parallel to the fibre axis. Figure 6a shows a schematic representation of the experimental setup, which involved rotating a permanent magnet with a 20 mT magnetic field strength around the Z axis. The cross-sectional area of the permanent magnets used was less than the sensing region of the STSCS. When the STSCS optical fibre magnetic sensor was initially placed in the centre between the two magnets, the spectral dip was observed to slowly shift from its initial position towards a longer wavelength before reaching a stable value after 2 min. This initial drift occurs because the magnetic field created by the permanent magnets is strongest at the centre of the STSCS, attracting the FMPs in the MF towards the centre and causing a small change in RI. When the concentration of FMPs around the STSCS becomes stable, no further change in RI occurred while rotating the magnetic field direction along the X–Y plane. Figure 6b show the results of this experiment (after the spectral dip had stabilised). Since the magnetic field direction is always perpendicular to the STSCS axis in this configuration, neither the particle density nor the RI change. As a result, the wavelength does not shift as the magnetic field is rotated in the X–Y plane.

Figure 7c shows the experimental results of rotating the permanent magnets along the X–Z and Y–Z planes. The FMPs gather along the surface of the capillary tube, creating a multiparticle chain when the magnetic field is applied to the STSCS fibre sensor encapsulated in the MF. Furthermore, their density surrounding the STSCS starts to become asymmetrical and nonhomogeneous with respect to the fibre axis. When the magnetic field direction is rotated in the X–Z or Y–Z planes, the direction of the FMP chains and their density around the STSCS also rotate, affecting the RI of the MF surrounding the STSCS, ultimately affecting the spectral response of the sensor. When α was set to 0° (i.e., the magnetic field axis was parallel to the STSCS axis), the maximum wavelength shift was observed, while the minimum wavelength shift was observed when α was set to 90° (i.e., the magnetic field axis was perpendicular to the STSCS axis). Due to the tapered shape of the STSCS, the maximum wavelength shift in the X–Z plane was greater than that in the Y–Z plane.

## 5. Conclusions

A high-sensitivity magnetic field sensor based on STSCS optical fibre structure encapsulated in a MF was proposed and investigated. By analysing the effect of the taper waist diameter of the STSCS and the concentration of the MF surrounding it, the magnetic sensitivity of this sensor was examined. The magnetic sensor showed reversible results, with a maximum sensitivity of 0.46 nm/mT for an STSCS with a 10 μm taper waist diameter, encapsulated in 1.22% FMPs in the MF. The influence of the magnetic field orientation along the X–Y, X–Z, and Y–Z axes was also investigated for developing a vector magnetic field sensor. The proposed sensor had a response time of 2 min, which is necessary for FMPs to release residual magnetism and produce a stable response. Therefore, the sensor may not be suitable for the real-time monitoring of magnetic field strength, but such an optical-fibre-based sensor has the advantage of immunity towards electromagnetic interference, and it can be used in hazardous environments, such as nuclear power plants and cyclotrons.

## Figures and Tables

**Table 1:** Sensitivity values for the STSCS optical fibre sensor of 10, 15, and 20 μm diameter surrounded by 0.39, 0.81, and 1.22% FMPs in the MF.

% of FMPs in MF	Sensitivity (nm/mT)		
	STSCS dia. = 20 μm	STSCS dia. = 15 μm	STSCS dia. = 10 μm
1.22	0.26	0.365	0.466
0.81	0.12	0.208	0.275
0.39	0.049	0.086	0.122

## Data Availability

Not applicable.
